# Management of dental caries lesions in patients with disabilities: Update of a systematic review

**DOI:** 10.3389/froh.2022.980048

**Published:** 2022-10-28

**Authors:** Gustavo Molina, Mariana Zar, Alison Dougall, Colman McGrath

**Affiliations:** ^1^ Division of Restorative Dentistry, Faculty of Dentistry, The University of Hong Kong, Hong Kong, Hong Kong SAR, China; ^2^ Department of Special Care Dentistry, The Dental School, Universidad Católica de Córdoba, Córdoba, Argentina; ^3^ Special Care Dentistry, Dublin Dental Hospital, Trinity College, Dublin, Ireland; ^4^ Applied Oral Sciences and Biomaterials, Faculty of Dentistry, Hong Kong University, Sai Ying Pun, Hong Kong SAR, China

**Keywords:** disabled persons, dental care for disabled, systematic review, caries prevention, restorative care

## Abstract

The aim of this systematic review was to update an existing review on the management of dental caries lesions in patients with disabilities so as to provide an up-to-date summary of the evidence. Randomized clinical trials and cohort studies related to preventive and restorative programmes for dental caries among people requiring special care, published in English, Spanish, Portuguese, French and German languages from February 1st 2011 to April 1st 2022, were retrieved from three databases (“updated review”). From the 1,105 titles identified using the search topic “Caries AND Disability”, 17 papers informed in the analyses: 6 referring to caries preventive strategies and 11 related to restorative care strategies. Most of these studies targeted children and adults with intellectual/physical disability, although preventive and therapeutic strategies were also reported for frail older adults and onchohematological patients. Fluorides in tablets, gels or varnishes forms and the use of xylitol as a sugar substitute were reported as effective approach to prevent the onset of caries in high-risk groups. Minimally intervention treatment options such as the Hall technique, the ART approach and the use of SDF for arresting caries, were deemed suitable and effective strategies for treating existing lesions in-office. In conclusion, in the past decade (2011–2022) an increased number of articles reported strategies to prevent and manage caries among people requiring special care. Although an array of preventive and therapeutic strategies for dental caries exists, more and better-quality clinical evidence is needed to offer guidance to inform policy and practice for special care dentistry.

## Introduction

In 2011, a systematic review regarding strategies to prevent and/or treat caries lesions in patients with disability concluded that more studies, and specifically more high-quality research was required to provide stronger scientific evidence to inform feasible and effective approaches to safeguard and improve the oral health of people requiring special care dentistry (1). The publication was a call for action to clinicians, researchers, and international oral health associations to spearhead the promotion of oral health, based on scientific evidence, for people with disability and medically compromised patients. Although the prevalence of caries is not necessarily higher among people with disabilities compared to the general population, it has been repeatedly described that people with disabilities have a higher number of untreated lesions, thus a higher unmet caries treatment needs as well as poorer oral hygiene and poorer periodontal status conditions than people without disabilities (2, 3).

In the last decade, in dentistry, major technical advances have led to improved quality of dental treatment and improved ability to maintain oral function and aesthetics over the life span. However, there are still gross and unfair inequalities in term of the quantity and the quality of dental treatment provided to people with disabilities. Special Care Dentistry is often perceived as the discipline of compromise—where “better than nothing” is the bottom line (4).

There is evidence that most but not all dental needs could be met in primary dental care settings (5). The problem for oral health care providers and governments is how to identify and select those who are best manged in the primary dental care setting and to decipher who needs additional specialist skills and adjuncts to receive and tolerate dental care. Furthermore, there is a need to ensure that such dental care is optimized and personalized to the patient's specific dental needs, considering that the scope of disability extends to a wide range of medical conditions that may compromise a “one-fits-all” approach of guidelines. Notwithstanding these considerations, references of best practices -targeting groups with similar characteristics- may help clinicians to offer their people with disabilities a variety of strategies that may suit them best to safeguard and improve their oral health.

The aim of this review was to update an existing review (1) on the management of dental caries lesions in patients with disabilities so as to provide an up-to-date summary of the evidence.

## Materials and methods

PICO principle (Population, Intervention, Comparator, Outcome) was used to define the research question: What is the suitability and effectiveness of available strategies for preventing and/or treating caries lesions in people with disability?

Three electronic databases, the Cochrane Library database for Clinical Trials, PubMed and LILACS (Latin American and Caribbean Health Science Literature) were searched, and all publications listed in the databases from February 1st 2011 to April 1st 2022 were included. Different combinations of MeSH terms, limits and Boolean operators were tested to identify those that could include the highest number of relevant publications. The search strategy repeated the one that had been used in 2011 with a change in the dates for the search instead. Details of the search strategy for each database are described in Appendix 3.

Reference-linkage of review articles were used to identify additional relevant publications. In addition, hand searches of key publications were undertaken to identify other studies that had not been retrieved from the electronic databases search.

The inclusion criteria were with respect to three aspects:

1) *Types of studies*. Randomized controlled (clinical) trials and cohort studies on preventive and restorative intervention programmes published in English, Portuguese, Spanish, French and German languages were included. If only a relevant title without a listed abstract was available, a full copy of the article was obtained and assessed.
2) *Type of participants*. People of any age and gender, presenting any medical condition related to disability. The intention was to include publications having a control group, either with or without disability. Definition of a person with disability was adopted from the WHO-ICF (International Classification of Functioning, Disability and Health) (6) referring to people who experience the negative aspects of the interaction between their environmental and personal context and any functional impairments, activity limitations and participation restrictions that they may present (6).
3) *Type of interventions*. Preventive and/or restorative intervention programmes for managing dental caries including but not limited to the use of chemical products to control cariogenic bacteria, remineralizing agents, restorative and non-restorative options for caries treatment. Case reports, narrative reviews and epidemiological studies were excluded for their analysis.

Titles and abstracts were initially retrieved in duplicate by 2 reviewers (GFM and MZ) to identify potentially included studies, discussing eligibility until agreement was achieved by consensus. Data regarding authorship, study design, type of intervention and outcomes of the interventions were extracted from the articles independently and in duplicate and recorded into an excel spreadsheet.

Studies were assessed by two reviewers (GFM, MZ) and double-checked by the other two review authors (AD, CM). Disagreements among the reviewers were resolved through discussion until agreement was reached. Two reviewers (MZ, GFM) independently assessed the risk of bias of included RCTs using the Cochrane Collaboration's tool (7) and disagreements were resolved by consensus. Each study was judged and categorized as being of low, moderate, high or unclear risk of bias. Unclear risk of bias was assigned to indicate lack of information or uncertainty about the potential for bias.

GRADE criteria (Grading of Recommendations Assessment, Development, and Evaluation—GRADE approach) (8) was used to rate the certainty of the evidence, based on the assessment of the study design, risk of bias, imprecision, inconsistency, indirectness, and magnitude of effect of the articles.

Finally, included articles were categorized regarding the hierarchy of the level of evidence (9) (Appendix 2), a tool that had been used in the systematic review that is being updated in this article, to analyze whether the findings of this review have improved the quality of the publications compared to those articles retrieved in the previous review (1).

To report the outcomes of this systematic review the guidance from the PRISMA Checklist (10) (Appendix 1) was followed.

### Analyses of data

Data were grouped in tables with a synoptic description of type of study, type of intervention and main outcomes to assess the relative effectiveness of each intervention. If studies reported dissimilar follow-up times or lacked a common comparator or if pairwise meta-analysis was not possible or failed to obtain measures of association, these data were reported as described by the primary study authors.

Quantitative analysis had been proposed by using Review Manager 5.3 software to compare studies using the same interventions and assessing the same outcomes by the same measurements. Meta-analysis were considered unfeasible for most of the outcomes due to the heterogeneity of the type of studies, participants and interventions, with a low number of strategies suitable for comparisons. A graphic showing the level of bias associated with each domain was obtained using this Cochrane software (Appendix 4).

Fixed effect model was applied if the total number of studies included in meta analysis was less than six studies (11). Due to lack of correlation in change from available studies, anticipated correlation of 0.5 was used to estimate the standard deviation of change (12). Continuity correction of 0.5 was applied if standard error cannot be computed due to estimated proportion is at 0 or 1 (13).

Finally, one-way ANOVA was used to analyze 5-year survival percentages among different restorative treatments.

## Results

A flow diagram of the systematic search is presented in Appendix 5. After reaching an agreement on potential suitable titles/abstracts, 52 full articles regarding preventive and/or therapeutical caries management strategies were retrieved for analysis of which 35 were finally excluded. Reasons for exclusion were: (1) articles describing epidemiological studies (*n* = 8); (2) narrative articles (*n* = 10); (3) case reports (*n* = 7); (4) articles not related to the topic of this review (*n* = 8) and (5) systematic reviews (*n* = 2).

A total of 17 publications were suitable for final analyses: 6 related to caries-preventive programmes and 11 to restorative treatment programmes.

### Caries-preventive strategies

Table 1 provides a summary of key information obtained from the included studies on caries preventive and therapeutic strategies. The level of the evidence of the included studies was rated between grades II and III-2, having included four RCTs (14–17), one cluster RCT (18) and one longitudinal field trial (19). The sample size of these six studies presented a significant variation among them with an impact on to the quality of the evidence. A total number of 532 participants was followed up in 6 clinical trials, in most cases recruiting primarily children and adolescents with intellectual disability (ID), with the exception of one study that was conducted in a population with visual/hearing impairment.

**Table 1:** A summary of key information obtained from the included studies regarding preventive and therapeutic strategies for caries management in people with disabilities.

Authors/year of public	Type of study	Type of participants	Type of intervention	Outcomes	Risk of bias	Hierarchy of the study
**Preventive strategies for caries management in people with disabilities**						
Mun et al, 2014	RCT (3-month intervention)	73 persons with intellectual disability	Three groups comparing oral hygiene, salivary flow and caries activity to assess a newly designed dental hygiene care programme using flash-based video, brochures and a toothpick method implemented by Dental Hygienists	Plaque index significantly decreased after each session in all three groups, and significant differences were found between groups (*p* = 0.036). Patients’ oral dryness decreased significantly, but stimulated saliva and dental caries activity did not improve.	High	III-1
Fenning et al, 2022	RCT (6-month intervention)	119 children with ASD (autistic spectrum disorder) 3–13 years old	Efficacy of parent training (PT) for improving oral hygiene and oral health	Compared with the toolkit intervention, PT was associated with increased twice-daily toothbrushing at 3 and 6 months and a reduction in plaque at 3 and 6 months. Comparatively fewer caries developed in children receiving the PT intervention over 3 months	Moderate	III-1
Watthabasaen et al, 2017	Cluster RCT (1-year intervention)	93 visual/hearing impaired students 7–18 years old	Xylitol chewing gums	Reduction of caries rate in primary dentition compared to control group; No differences in permanent dentition; Increase of remineralization; Improvement in oral hygiene.	Moderate	III-1
Baygin et al, 2014	RCT (1-year follow up)	90 non-cooperative patients that required intervention under GA 3–17 years old	Three groups comparing: (1) 0.1% fluoride varnish; (2) 1% chlorhexidine- 1% thymol varnish + 0.2% chlorhexidine-0.2% sodium fluoride; (3) toothbrushing only	The use of fluorides and chlorhexidine compounds help to reduce bacterial counts thus reducing the risk for caries and gingival problems	Moderate	II
Liu et al, 2013	RCT (2-year follow up)	103 school children with multiple disabilities 6–12 years old (mean 9.4 SD 1.8)	Fluoride tablets program in a non-fluoridated country	After 24 months, DMFT scores increased significantly less than the control group	Moderate	II
Patil et al, 2011	Longitudinal field trial	54 children with mild to moderate ID 7–17 years old	Individualized preventive program based on the Cariogram	44%–87% increase of chances to avoid caries after 10 months of implementation	High	III-2
**Therapeutic strategies for the management of caries in people with disabilities**						
Mendiratta et al, 2021	RCT (6-month follow up)	82 participants with ID Mean 15.2 and 17.6 years old	Comparison of the efficacy of SDF vs. Glass Ionomer + Fluoride varnish to arrest caries 2–3 Nyvad classification in permanent posterior teeth	CAR: SDF 94.5% vs. GI + NaF 90.1% No significant differences: the two strategies appeared successful at 6-month follow up	Moderate	II
Tong et al, 2021	Cohort study (5-year follow up)	846 frail older adults (mean 86.8 SD 10.5 years old) 3,201 restorations	Direct bonded restorations: RC vs. GIC in permanent teeth	Overall survival: 60.3% RC: 60.5% vs. GIC: 59.4% Moderate longevity of restorations with no statistical differences between materials	N/A	III-2
Ekstrand et al, 2013	RCT (8-month follow up)	125 frail older adults living in nursing homes (mean 81.7 SD 11.6 years old) At least 1 root caries lesion	Control of the progression of root caries lesions using 5,000 vs. 1,450 ppm fluoride toothpastes	5,000 ppm toothpaste is significantly more effective to control the progression of root caries lesions	Moderate	II
Robertson et al, 2020	Cohort study, prospective service evaluation (87 ± 24 months follow up)	16 children with learning disability (LD) 27 restorations (hall technique crowns)	Hall technique to restore primary molars	Survival: 100% Acceptability: 80% Satisfaction: 96%	N/A	III-3
Chang et al, 2017	Cohort study, retrospective evaluation of interventions (32.7 ± 20.0 months follow up)	203 non-cooperative patients ≥12 years old (mean 27.0 SD 14.1) 381 treated teeth included 267 were followed up	Single-visit Endodontic Treatment + Restoration under GA	5-year survival rate: 89.8% 5-year success rate: 86.4%	N/A	III-3
Maes et al, 2021	Cohort study, retrospective evaluation of interventions (5-year survival)	101 non-cooperative patients with ID and/or physical disability (PD) (Mean 37.3 SD 13.1 years old) 728 restorations	Direct RC restorations placed under GA (non-endodontically treated teeth)	5-year survival rate: 67.7%	N/A	III-3
Molina et al, 2015	RCT (1-year follow up)	66 patients with ID and/or PD (mean 13.6 SD 7.8 years old)	Atraumatic Restorative Treatment (ART/GIC) in office; Conventional Treatment (CRT/RC) in office and CRT under GA	ART was feasible/acceptable for 47 patients (79%); CRT in office was feasible for 5 (33%). CRT under GA was the only option for 14 patients (21%). Respondent satisfaction was higher for those receiving ART than CRT (in the clinic and under GA)	Moderate	III-1
Molina et al, 2019	RCT (5-year follow up)	66 patients with ID and/or PD (Mean 13.6 SD 7.8 years old) 298 restorations in primary and permanent teeth	Atraumatic Restorative Treatment (ART/GIC) in office; Conventional Treatment (CRT/RC) in office and CRT under GA	5-year survival: ART/GIC 90.2%; CRT/RC 89.8% Single surface ART/GIC 94.2%; CRT/RC 93.8% Multiple surface ART/GIC 76.4%; CRT/RC 61.8%	Moderate	III-1
Goncalves et al, 2015	Pragmatic controlled clinical trial (1-year follow up)	24 children undergoing oncohaematological treatment 101 ART restorations 14 healthy controls 52 ART restorations 3–13 years old	ART restorations and sealants in single surfaces of primary and permanent molars	High occurrence of failures in need to repair. This treatment needs to be closely followed up.	High	III-2
Pani et al, 2018	Retrospec-tive cohort study (2-year follow up)	87 non-cooperative children that required GA (Mean 47 SD 7.2 months old) 254 restorations in primary teeth	RC vs. HVGIC restorations in single surfaces of primary teeth under GA	High failure rates, very low survival with no statistically significant differences between the two restorative materials	N/A	III-3
Mallineni et al, 2018	Retrospec-tive audit	275 non-cooperative patients (Mean 12.4 SD 10.2 years old)	Most prevalent dental treatment performed under GA	RC and Stainless-steel crowns are the most frequent restorations in primary teeth Amalgam is the most frequent restorative material used in permanent teeth. No survival data.	N/A	III-3

There was a significant heterogeneity in the type of interventions and conclusive results were of low certainty in most of studies.

Three studies introduced strategies that focused on how to effectively produce changes in attitudes and behavior towards oral health care, implemented in people with intellectual disability and autistic disorder spectrum. The study by Mun et al. (14) assessed the effectiveness of a dental hygiene care programme implemented by Dental Hygienists in a population with intellectual disability whereas the study by Fenning et al. (15) emphasized the importance of training the parents to improve oral hygiene standards in children with autistic spectrum disorders. Oral health variables such as plaque index or caries activity measured the impact of these strategies, reporting promising results for targeting special groups with individualized and creative methods (14, 15).

The third of these types of studies, followed the line of assessing caries risk to target individualized strategies to prevent the onset of the disease. The Cariogram assessment model (20) was adopted to follow up customized preventive programs in a population of 54 children with ID, adjusting different risk factors according to each child's situation to avoid the development of new lesions (19). Despite promising results of this aiding tool, details of specific strategies to modify the individuals’ caries risk were not provided in this study.

Two studies referenced the effectiveness of chemical agents for controlling cariogenic biofilm, confirming the evidence that fluorides in different concentrations and presentations (tablets, gels, varnishes and pastes) may be useful resources for preventing caries lesions (16, 17). Although chlorhexidine has been mentioned in one of these studies (17), the evidence of its effectiveness has of low quality and uncertain.

The use of xylitol chewing gums in a visual/hearing impaired school population was a successful strategy to relent DMFT scores after 1 year (18). More studies are needed to confirm of reject these findings in order to promote a generalized use of these sugar substitutes to prevent the onset of caries in people with disability.

### Restorative/non-restorative treatment strategies

Table 1 provides a summary of key information obtained from the included studies on restorative treatment programmes. The quality of the included studies rated between level II and lV. Three RCT (one of them has two entrances for different types of outcomes) (21–24), one pragmatic controlled clinical trial (25), one prospective cohort study (26), one retrospective audit (27), and four retrospective cohort studies (28–31) reported the outcomes of different interventions that aimed to restore teeth affected by caries disease in patients with disability in time interval 2011–2022.

The use of minimally invasive strategies had a double purpose in this population: (1) maximal preservation of tooth structures and (2) to avoid interventions under GA as much as possible.

Following a gradient of complexity of the interventions, two studies reported that fluorides in high concentrations have shown to be effective products to arrest root caries lesions (22) as well as to prevent the progression of occlusal lesions in permanent molars, either using SDF or a combination of fluoride varnish sealed with a glass ionomer restorative cement (21). Although the two studies evaluated the ability to arrest the progression of caries lesions, they used different criteria to report caries arrestment rates, allowing no comparison between the outcomes obtained for each study.

The ART approach, employing high-viscosity glass ionomer cement as a restorative material, was tested in two of the included studies, seeking for alternatives to conventional restorative treatment that comprises the use of rotary instruments for caries removal. One of these clinical trials reported 76% acceptance and 100% satisfaction with this treatment modality in one article (23) and overall survival of 90.2% after 5 years in a separate paper (24). The other article on ART targeted a population of children undergoing oncohaematologic treatment and, although it was stated that this approach is suitable for this group of patients, a high number of restorations in need for repair was detected in a short period (1-year follow up) suggesting that these restorations needed a close and frequent follow up (25).

The decision of using either resin composite (RC) or glass ionomer (GI) as the restorative material of choice was also analyzed in a cohort study that evaluated survival of direct bonded restorations in frail older adults (30). Results showed no statistically significant differences between these two materials and overall survival rates of 60.5% after 5 years (30).

To overcome low survival rates of large restorations, another minimally invasive resource in this review was the Hall technique to arrest caries lesions in primary teeth of 16 children with learning disability. This prospective cohort study reported 100% survival, 80% acceptance and 96% satisfaction respectively (26).

A retrospective audit of interventions undertaken aided by GA revealed that resin composite restorations and stainless-steel crowns are the most frequently used resources to restore teeth affected by caries in the primary dentition whereas dental amalgam is the preferred restorative material chosen for the permanent dentition (27).

As regards to restorative treatment carried out under GA, high failure rates were observed in single surface restorations performed in primary molars under GA in one study (29). Another retrospective study carried out in the permanent dentition of patients with intellectual and/or physical disability reported 67.7% survival after 5 years of follow up (31). These results were obtained in non-endodontically treated teeth, whereas 89.8% survival and 86.4% success were reported for single-visit endodontic treatment and restoration in permanent teeth that received treatment under GA (28).

Finally, irrespective of the heterogeneity of the reports, a comparison of outcomes regarding survival of different types of restoration at different follow-up periods, determined that the Hall technique was 100% effective at an average of a 2-year period. No significant difference after 5 years was observed for ART/HVGIC, resin composite (RC) and root canal treatment followed by single-visit RC restoration in permanent teeth (90.2%, 89.8% and 89.8% respectively; *p* ≥ 0.05). Results obtained after 5 years were significantly different for resin composite restorations in two studies, being 89.8% (21) and 67.7% (28) respectively, the latter placed under GA exclusively.

With respect to multiple-surface restorations, similar results were obtained in two studies (24, 30) for glass ionomer cement (GIC) and RC after 5 years, ranging from 59.4% to 76.4% (*p* = 0.06). Standard deviations of these percentages explain there were no statistical differences among these outcomes.

The lowest percentages of survival were observed for restorations in primary teeth, either using GIC or RC.

### Certainty of the evidence

The analyzes of the certainty of the evidence using GRADE criteria for outcomes across studies is summarized in Table 2. Five different outcomes were identified for the prevention of caries and two outcomes for therapeutic strategies in the studies that had been included. The number of studies per outcome and the heterogeneity of these studies allowed few statistical analyses, leading to weak recommendations based on the power of this evidence.

**Table 2:** Analysis of the certainty of the evidence by outcomes across studies based on GRADE criteria.

Outcome	Anticipated absolute effect (95% CI)	Relative effect (95% CI)	Number of participants (studies)	Certainty of the evidence (GRADE)	Comments
**Preventive strategies for caries management in people with disabilities**					
Reduction of plaque index (PI)	−0.02	(−0.07, 0.03)	282 (3 RCT)	⊕⊕⊝⊝ Low^a^	Populations with similar characteristics
Reduction on bacterial counts	Not suitable for comparisons		90 (1 RCT)	⊕⊕⊝⊝ Low^b^	Only one study that measured this outcome
Reduction of caries experience (DMFT)	−1.62	(−1.86, −1.38)	468 (2 RCT)	⊕⊕⊕⊝ Moderate^c^	Results obtained at 6 and 24 months
Reduction of caries experience (DMFS)	−1.35	(−1.78, −0.92) (within 2 years)	523 (2 RCT)	⊕⊕⊕⊝ Moderate^d^	Results obtained at 12 and 24 months
Chances to avoid caries	Not suitable for comparisons		54 (1 LFT)	⊕⊝⊝⊝ Very low^e^	Preventive impact of this strategy is based on indirect outcomes
Adverse effects	No adverse effects are reported regarding preventive strategies outcomes in these studies				
**Therapeutic strategies for caries management in people with disabilities**					
Caries Arrestment Rate (CAR)	86%	(82%, 89%) (within 8 months) (RCT only)	223 (2 RCT) 17 (1 CS)	⊕⊕⊝⊝ Low^f^	Non-restorative strategies were reported with very good results
	88%	(84%, 91%) (within 2 years) (All)			
Survival of the restorations	87%	(83%, 91%) (within 5 years) (RCT only)	66 (1 RCT) 38 (1 PCT) 1253 (5 CS)	⊕⊕⊝⊝ Low^g^	Heterogeneity in types of restorations, in type of participants and in types of studies
	72%	(58%, 85%) (within 5 years) (All)			
Adverse effects	No adverse effects are reported regarding therapeutic strategies outcomes in these studies				

CI, confidence interval; RCT, randomized controlled trial; LFT, longitudinal field trial; PCT, pragmatic controlled trial; CS, cohort studies; DMFT-dmft, decayed, missing, filled teeth in permanent and in primary dentition. Negative values in the reduction implied the increase in the outcome after the treatment.

^a^ Downgraded two levels due to outcome reported in three RCTs, one with high and two with moderate risk of bias.

^b^ Downgraded one level due to outcome reported only in one RCT (magnitude of the effect) and one level due to moderate risk of bias.

^c^ Downgraded one level due to heterogeneity in study designs.

^d^ Downgraded one level due to heterogeneity in study designs.

^e^ Downgraded three levels, one due to the very low magnitude of the effect, one due to indirectness of the outcome and one due to high risk of bias.

^f^ Downgraded two levels, one due to heterogeneity in study designs and one due to low magnitude of the effect.

^g^ Downgraded two levels, one due to heterogeneity in study designs (1 RCT, 1 PCT and 5 CS) and one due to moderate risk of bias in the only RCT for this outcome.

## Discussion

Diversity is a distinctive feature that relates to the population of people with disability. The broad spectrum of medical conditions that are included within the scope of disability (32) creates a context of heterogeneity that makes it difficult to provide clear recommendations that might be suitable for the whole spectrum. However, if we addressed that the etiology of caries disease is universal, meaning that it may affect any person that ticks the necessary boxes of risks factors, we may assume that preventing caries would be a matter of avoiding such risks irrespective of any medical condition. The type of disability itself may comprise specific risks that must be disclosed and taken into consideration when planning a preventive programme (33). That seems to have been acknowledged in some of the studies retrieved for this review.

Recent systematic reviews that analyzed strategies for oral care in people with disabilities show that the introduction of different strategies to reduce the risk for the development of caries disease had been reported in clinical studies comparing their efficacy, such as the use of special/modified manual vs. electric or powered toothbrushes, oral hygiene training of carers and of people with disability and the frequency of supervised toothbrushing, as well as the impact of regularly scheduled visits plus supervised toothbrushing and the discussion of photographs as motivators for oral care (34, 35). Beyond the use of universal strategies that have proved to be effective for the general public, like the use of mechanical and chemical resources to control the cariogenic biofilm, insights of contextual (training of carers) and personal (training of patients, discussion of photographs as motivators) factors have been brought to the table to tackle the problem in daily actions that may successfully and sustainably prevent the onset of caries in this at-risk population.

Although the number of publications regarding preventive strategies for managing caries in people with disability included in the present review is somewhat low, noticeable is the trend of interdisciplinary approach in comparison to the updated review. The evaluation of these resources is highlighted as a new perspective for the prevention of the disease as such, irrespective of the use of specific products, focusing on a more up to date concept of caries and the factors that trigger the onset of the disease instead of the mere avoidance of cavities. In this regard, there seems to be a shift from -for instance- “using fluorides” to “how we introduce an effective and sustainable use of fluorides according to the characteristics of each group of patients within the spectrum”. Far from being a semantic discussion, authors believe this is a key innovative approach for an old situation of unmet needs. Therefore, interdisciplinary studies involving social sciences may provide the frame for supporting strategies that may effectively reach these patients.

Unfortunately, when the disease has been activated in the oral environment, efforts must be taken to reverse the situation while treating the lesions that develop in such a condition. For that purpose, cooperation of the patient to undertake either restorative or non-restorative interventions is essential to achieve sustainable outcomes. Probably due to the barriers to provide restorative care to this population, most of the participants included in the studies on therapeutic strategies were some types of “non-cooperative” patients, including intellectual and/or physically to medically compromised patients, ranging in age from children to frail older adults. This means that a broad scope of situations was covered by the umbrella of the designs of these studies. However, the type of intervention evaluated in each study was mainly focused on the most prevalent dental treatment needs for each stage of their lifespan and therefore, generalized strategies for all stages were difficult to retrieve due to this heterogeneity.

The tooth restorative cycle speed seems to be increase exponentially in high-risk patients and, at the same time, risk factors may be too difficult to approach in certain medical conditions or in people with behavior problems. Non or minimally invasive techniques may help to introduce more patient-friendly techniques without compromising the quality of the interventions as it has been shown in clinical studies using SDF or the ART approach (36, 37). However, longevity of the restorations, regardless of using resin composite or glass ionomer, may not achieve minimum standards and therefore, need to seek for better alternatives. Interestingly, failures occur equally for restorations either performed in-office or under sedation or GA, meaning that a different type of restoration which may withstand worse challenges in the oral cavity must be sought. The use of indirect restorations may be one option, including CAD-CAM resources, should be explored as a part of the arsenal of strategies that might be offered to people with disability if quality treatment becomes an irrefutable aim.

Although a higher number of suitable articles have been included for analysis in comparison to the previous review, the present study presents almost the same limitations, as there is still a lack of well-designed randomized clinical trials regarding both preventive and restorative approaches appropriate for use in patients with disabilities. RCTs are the most appropriate type of studies for the evaluation of clinical treatments and the introduction of new or modified dental materials prior to marketing (38). The control of bias and the equal distribution of known confounding factors between groups are among many advantages that this kind of study provides. However, some of its obvious disadvantages are related to the fact that it might take a long time to obtain outcomes and possibly a long time to enroll patients (9). Moreover, obtaining approval in Ethics Committees may discourage researchers for these endeavors or extend the times for developing the proposals extensively, leading to incomplete or low-quality protocols usually abandoned halfway.

In Disability and Oral Health, reports of clinical interventions are commonly restricted to isolated cases or case series that represent a lower level of evidence for their inclusion in a systematic review. This is a limiting aspect that has a significant impact on the construction of evidence-based guidelines for best practices in Special Care Dentistry. This issue has been discussed in the search of evidence for the use of dental implants among people with disabilities, but such controversies may also apply to Operative Dentistry as is the case of this review (39). Once again, these facts call for action. International organizations such as the International Association for Disability and Oral Health (IADH), the Special Care in Dentistry (SCD), the IADR, FDI and WHO, should give greater priority to the oral health of people with disabilities and measure core outcomes that are meaningful to them. Research methodology should be included or made mandatory in specialization courses for special needs dentists. Importantly, guidance should be provided regarding the development of an international agenda of relevant research topics and inclusive exclusion criteria should be scrutinized within ethics applications of ALL research studies to ensure that people with disabilities are only excluded with good reason in research studies which should reflect the diversity of the community (40). Furthermore, in view of the significant numbers of people with disabilities worldwide -over 1 billion people live with some form of disability; the number of people with disability are dramatically increasing due to demographic trends and increases in chronic health conditions, among other causes- (41), epidemiological data should always include those with disabilities and other hard to reach groups within their data collection. It is with great pleasure to notice that guidelines for carrying out research and for treating people with disability have been placed on the agenda of the forthcoming congresses of the IADH and SCD.

## Conclusion

In the past decade (2011–2022) new evidence regarding the suitability and effectiveness of preventive and therapeutic strategies for managing caries lesions in people with disabilities has been published. There seems to be a shift in the focus of preventive strategies by introducing resources to effectively target special groups depending on their characteristics. For the restorative approach, minimally invasive strategies such as the use of SDF, ART and the Hall technique have demonstrated to be suitable and effective for the treatment of caries lesions both in primary and permanent teeth in office, thus reducing the need for interventions under general anesthesia. This review offers guidance to inform policy and practice for special care dentistry.
