# Supplementary material for: Management of dental caries lesions in patients with disabilities: Update of a systematic review
Source: Front Oral Health. 2022 Oct 28;3:980048. doi: 10.3389/froh.2022.980048 (PMC9650433; doi:10.3389/froh.2022.980048)
Supplement: Supplementary file 2 [file Datasheet2.pdf]

Appendix 2 Criteria for assessing the level of evidence of included publications

Level of Evidence	Description
I	Systematic review of level II studies
II	Randomized controlled trial (RCT)
III-1	A pseudo-RCT (alternate allocation of some other method)
III-2	A comparative study with concurrent controls; non-randomized experimental trial; cohort study, case-control study; interrupted time series with a control group
III-3	A comparative study without concurrent controls; historical control study; two or more single arm studies
IV	Case series with either pre-test / post-test outcomes
