# Supplementary material for: Management of dental caries lesions in patients with disabilities: Update of a systematic review
Source: Front Oral Health. 2022 Oct 28;3:980048. doi: 10.3389/froh.2022.980048 (PMC9650433; doi:10.3389/froh.2022.980048)
Supplement: Supplementary file 3 [file Datasheet3.pdf]

Appendix 3. Search strategy

1. In PubMed

   Initial search using “Caries AND Disability”

   Total: 1066 titles

   Different combinations of MeSH terms, limits and Boolean operators were tested, to define those that could include the highest number of relevant publications.

   The final strategy used was:

   "Dental Caries/therapy"[Mesh] AND ("Dental Care for Disabled"[Mesh] OR "Disabled Persons"[Mesh] OR "Disabled Children"[Mesh] OR "Mentally Disabled Persons"[Mesh] OR "Communication Aids for Disabled"[Mesh]) NOT ("Periodontal Diseases"[Mesh] NOT "Surgery, Oral"[Mesh] NOT "Editorial"[Publication Type] NOT "Letter "[Publication Type] NOT "Case Reports"[Publication Type] NOT "Review"[Publication Type]) AND (hasabstract[text] AND (Clinical Trial[ptyp] OR Randomized Controlled Trial[ptyp] OR Clinical Trial, Phase I[ptyp] OR Clinical Trial, Phase II[ptyp] OR Clinical Trial, Phase III[ptyp] OR Clinical Trial, Phase IV[ptyp] OR Controlled Clinical Trial[ptyp] OR English Abstract[ptyp] OR Journal Article[ptyp]) AND (English[lang] OR French[lang] OR German[lang] OR Spanish[lang] OR Portuguese[lang]) AND ("2011/02/01"[PDAT]: "2022/04/01"[PDAT])).

   Total: 32 titles

2. In Lilacs

   Terms “Caries” AND “Disability” were used to search for relevant publications

   Total: 80 titles

3. In the Cochrane Library database for randomized clinical trials

   Terms “Caries AND Disability” ------≥ search for trials ------≥ filtered by “years” from 01/02/2011 to 01/04/2022

   Total: 66 titles
