# Supplementary material for: Management of dental caries lesions in patients with disabilities: Update of a systematic review
Source: Front Oral Health. 2022 Oct 28;3:980048. doi: 10.3389/froh.2022.980048 (PMC9650433; doi:10.3389/froh.2022.980048)
Supplement: Supplementary file 4 [file Datasheet4.pdf]

Appendix 4. Risk of bias assessment graph – Domains that were considered for the evaluation of the selected articles using Reviewer Manager 5.3

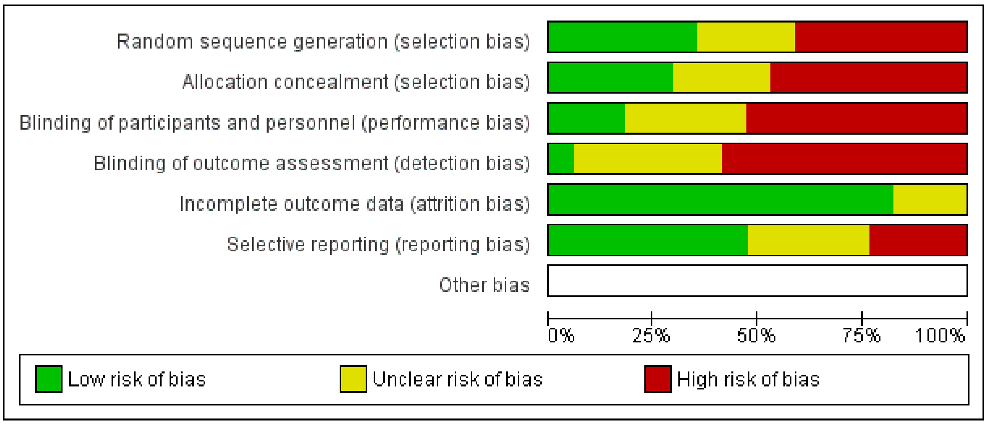

Caption

Risk of bias graph: review authors' judgements about each risk of bias item presented as percentages across all included studies.
