# Supplementary material for: Management of dental caries lesions in patients with disabilities: Update of a systematic review
Source: Front Oral Health. 2022 Oct 28;3:980048. doi: 10.3389/froh.2022.980048 (PMC9650433; doi:10.3389/froh.2022.980048)
Supplement: Supplementary file 5 [file Datasheet5.pdf]

Figure 1- PRISMA Flow chart of articles included for the study

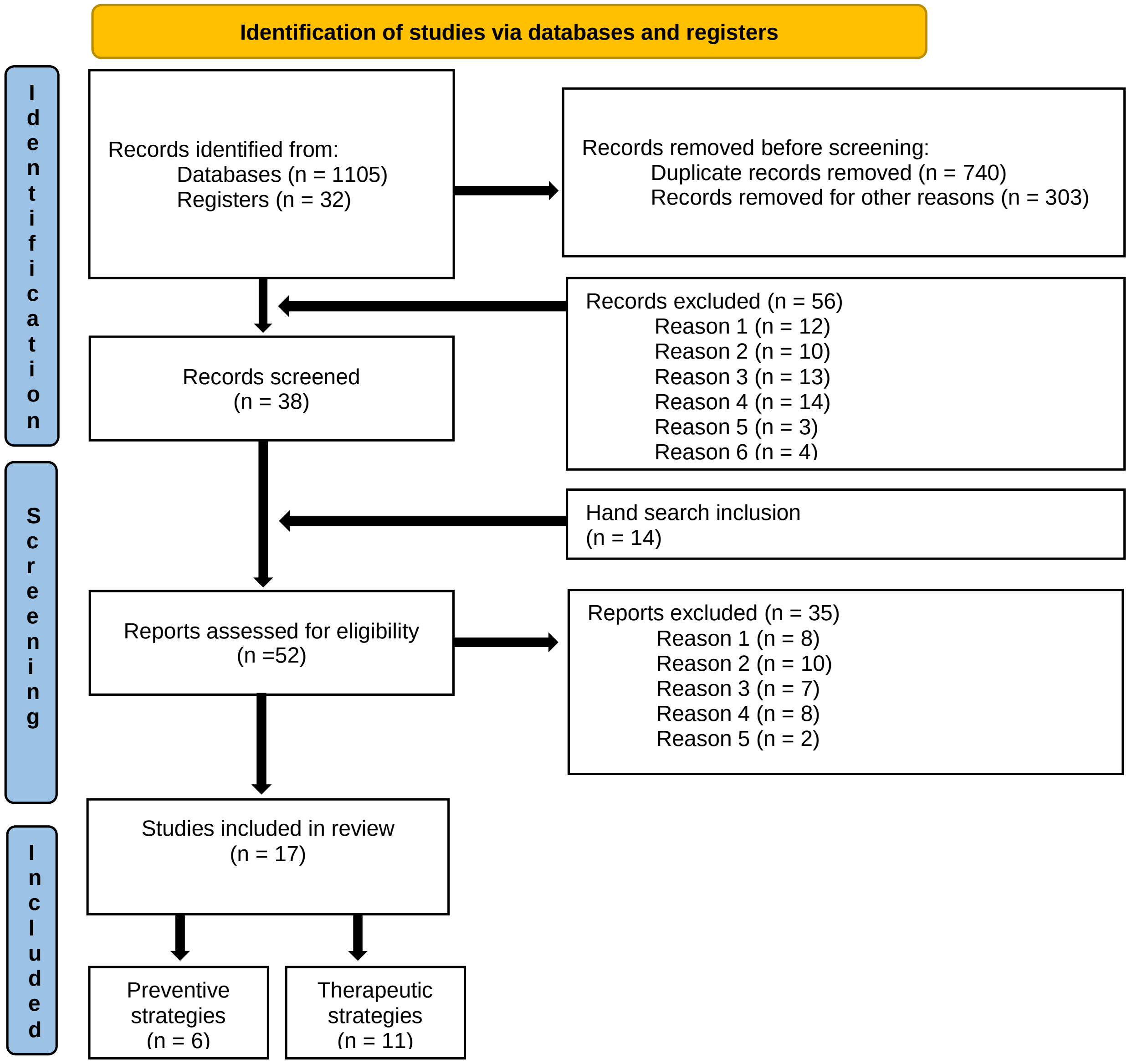

Reasons for exclusion:

- Reason 1 – Epidemiological studies
- Reason 2 – Narrative articles
- Reason 3 – Case reports
- Reason 4 – Not related to the topic of this review
- Reason 5 – Systematic reviews
- Reason 6 – Results not published yet

From: Page MJ, McKenzie JE, Bossuyt PM, Boutron I, Hoffmann TC, Mulrow CD, et al. The PRISMA 2020 statement: an updated guideline for reporting systematic reviews. BMJ 2021;372:n71. doi: 10.1136/bmj.n71
